# Small-Cell Carcinoma of the Bladder With Concurrent High-Grade Prostate Cancer: A Case Report and Review of the Literature

**DOI:** 10.7759/cureus.111470

**Published:** 2026-06-25

**Authors:** Veronia Fahmy, Anna Huynh, Alec M Block, James S Welsh

**Affiliations:** 1 Radiation Oncology, Loyola University Medical Center, Maywood, USA; 2 Medicine, University of Texas Medical Branch at John Sealy School of Medicine, Galveston, USA; 3 Radiation Therapy, Edward Hines Jr. VA Hospital, Hines, USA

**Keywords:** bladder-preserving chemoradiotherapy, high-risk prostate adenocarcinoma, hypofractionated radiotherapy, mri surveillance, multimodal therapy, neuroendocrine bladder carcinoma, simultaneous integrated boost (sib), small cell carcinoma of the bladder, synchronous malignancy, volumetric modulated arc therapy (vmat)

## Abstract

Small-cell carcinoma of the bladder (SCCB) is a rare, aggressive neuroendocrine malignancy associated with early metastasis and poor survival. Due to its rarity, optimal management is not well defined and is generally extrapolated from small-cell lung cancer treatment paradigms. We report a rare case of SCCB with synchronous high-grade prostate adenocarcinoma managed with a bladder-preserving multimodal approach.

An 82-year-old man presented with hematuria and urinary retention, who was diagnosed with limited-stage SCCB following transurethral resection of bladder tumor (TURBT). He received neoadjuvant cisplatin and etoposide, but definitive therapy was significantly delayed due to severe necrotizing perineal infection with fistula formation requiring multiple surgical interventions. During this period, he was also diagnosed with high-risk prostate adenocarcinoma (Gleason 4 + 5 = 9, prostate-specific antigen (PSA) > 100 ng/mL, with normal PSA range less than or equal to 4 ng/mL) and was initiated on androgen deprivation therapy.

After recovery and surgical repair of a large inguinal hernia (that would have hindered external beam radiation therapy planning), he underwent definitive bladder-preserving radiotherapy using simultaneous integrated boost volumetric modulated arc therapy (VMAT), delivering 60 Gy in 20 fractions to the prostate and proximal seminal vesicles, 55 Gy in 20 fractions to the whole bladder, and 44 Gy in 20 fractions to the elective pelvic lymph nodes.

Treatment was well tolerated with minimal toxicity. Follow-up cystoscopy and imaging demonstrated a complete response in both malignancies. More than three years after treatment, the patient remains without evidence of disease, with a low PSA and no evidence of local, distant, or intracranial recurrence on serial brain magnetic resonance imaging (MRI) studies.

This case demonstrates that durable long-term disease control in SCCB may be achievable with individualized bladder-preserving multimodal therapy, even in the setting of significant treatment delays, severe intercurrent complications, and synchronous high-grade prostate cancer. It also highlights the feasibility of simultaneous integrated pelvic radiotherapy for dual malignancies and supports MRI surveillance as a reasonable alternative to prophylactic cranial irradiation in carefully selected patients with SCCB.

## Introduction

Small-cell carcinoma of the bladder (SCCB) is a rare and highly aggressive neuroendocrine malignancy, accounting for less than 1% of all bladder tumors and around 4% of all small-cell carcinomas (SCCs) [1-4]. Histologically, SCCB is largely indistinguishable from small-cell lung cancer (SCLC), demonstrating a strong male predominance (an approximately 5:1 M:F ratio) and typically presents in older patients, with a mean age of 66 years [4,5]. The disease is characterized by early metastatic dissemination; approximately 60% of patients have metastases at diagnosis [6], and outcomes are poor, with median overall survival (OS) ranging from 12 to 28 months depending on the stage at presentation, and five-year survival rates of approximately 16%-22% despite multimodal therapy [7,8].

SCCB shares key molecular features with SCLC, including near-universal inactivation of TP53 (tumor protein p53) and RB1 (retinoblastoma 1), as well as a high somatic mutational burden. However, the presence of alterations commonly associated with urothelial carcinoma, such as ARID1A (AT-rich interaction domain 1A), CREBBP (CREB-binding protein), and KDM6A (lysine demethylase 6A), supports a shared urothelial origin rather than a pulmonary lineage [9,10]. More recent transcriptomic analyses have identified distinct molecular subtypes defined by lineage-specific transcription factors, including ASCL1 (achaete-scute family bHLH transcription factor 1), NEUROD1 (neuronal differentiation 1), and POU2F3 (POU class 2 homeobox 3), which may have implications for prognosis and therapeutic targeting [11,12].

Given its rarity, no prospective randomized trials have established a standard treatment paradigm for SCCB. Current management is largely extrapolated from SCLC and supported by retrospective data. Contemporary guidelines for localized disease recommend platinum-based chemotherapy in combination with definitive local therapy, such as neoadjuvant chemotherapy followed by cystectomy or bladder-preserving chemoradiotherapy [13]. Multimodal approaches have demonstrated improved outcomes compared with single-modality treatment, with median OS exceeding 29 months for chemoradiation and up to 46-64 months in surgical series [14].

The role of prophylactic cranial irradiation (PCI) in SCCB remains controversial [15]. While PCI has been shown to reduce the incidence of brain metastases and improve survival in SCLC, the risk of intracranial metastases in SCCB appears substantially lower [16,17]. Retrospective series report rates ranging from approximately 1.5% to 10%, suggesting a different natural history compared with SCLC [18-20]. Accordingly, most guidelines favor surveillance with brain magnetic resonance imaging (MRI) rather than routine PCI, although this remains an area of ongoing debate [21].

Here, we present the case of a non-healing patient with SCCB and concurrent Gleason 9 (Grade Group 5) prostate adenocarcinoma with a markedly elevated prostate-specific antigen (PSA) level. Management was further complicated by a perineal fistula and a large inguinal hernia requiring surgical repair prior to definitive radiation therapy. Despite these challenges and the aggressive biology of SCCB, the patient achieved a durable complete response with a bladder-preserving multimodal approach and remains disease-free more than three years after diagnosis. This case highlights the potential for long-term survival in SCCB and contributes to the evolving discussion regarding the role of surveillance neuroimaging in lieu of PCI in this population.

## Case presentation

An 82-year-old man with Eastern Cooperative Oncology Group Performance Status (ECOG) 1 presented in 2022 with hematuria and urinary retention and was diagnosed with limited-stage SCCB following transurethral resection. Histopathology demonstrated SCCB. Immunohistochemical staining showed positivity for synaptophysin/chromogranin/CD56 and supported the diagnosis of neuroendocrine carcinoma. Staging imaging demonstrated no distant metastatic disease. He initiated neoadjuvant cisplatin and etoposide chemotherapy.

His clinical course was complicated by a severe necrotizing perineal infection with fistula formation requiring multiple surgical debridements, intravenous antibiotics, and prolonged rehabilitation, resulting in substantial delay in definitive pelvic radiotherapy. During this interval, he also required surgical repair of a massive inguinal hernia, further delaying oncologic treatment.

During recovery, laboratory evaluation revealed a markedly elevated PSA level (>100 ng/mL, with a normal PSA range less than or equal to 4 ng/mL). Prostate biopsy subsequently confirmed synchronous very high-risk prostate adenocarcinoma (Gleason score 4 + 5 = 9). Androgen deprivation therapy (ADT) was initiated and continued for 24 months. Interestingly, the treatment delays may have facilitated timely diagnosis and definitive management of the synchronous prostate malignancy.

**Figure 1:**
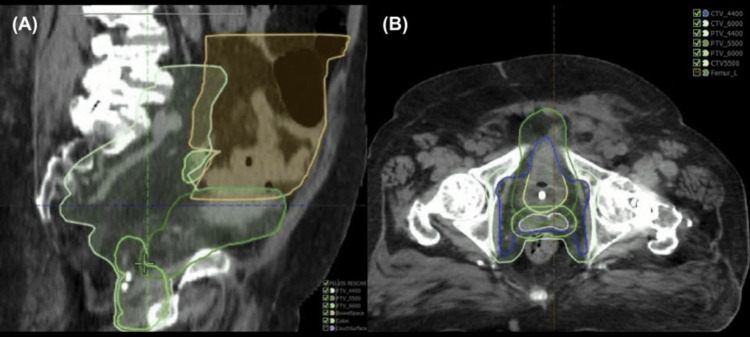
Images of target volumes for risk-adapted simultaneous integrated boost radiotherapy (A) Image of contoured planning target volumes (PTV). (B) Image of contoured clinical target volumes (CTV)

Following recovery from infection and surgical repair of the large inguinal hernia, the patient underwent definitive bladder-preserving radiotherapy using a simultaneous integrated boost (SIB)-volumetric modulated arc therapy (VMAT) technique. Treatment planning was further complicated by the presence of a left hip prosthesis, which affected dose optimization and image guidance. Radiation was delivered in 20 fractions to three dose levels simultaneously: 60 Gy to the prostate and proximal seminal vesicles (3 Gy per fraction), 55 Gy to the whole bladder (2.75 Gy per fraction), and 44 Gy to the elective pelvic nodal volume (2.2 Gy per fraction). Dose prescriptions and organ-at-risk constraints are summarized in Tables 1-3, with corresponding target volume contours illustrated in Figure 1.

**Table 1:** Radiation prescription for pelvic radiotherapy PTV: planning target volume

Structure	Total dose	Fractionation	Coverage goal
PTV_6000	60 Gy	20 fractions (3 Gy/fraction)	≥95% of PTV receives ≥100% of prescribed dose
PTV_5500	55 Gy	20 fractions (2.75 Gy/fraction)	≥95% of PTV receives ≥100% of prescribed dose
PTV_4400	44 Gy	20 fractions (2.2 Gy/fraction)	≥95% of PTV receives ≥100% of prescribed dose

**Table 2:** Techniques used for pelvic radiotherapy VMAT: volumetric modulated arc therapy; CBCT: cone-beam computed tomography

Technique	
Modality	6 MV photons
	VMAT (RapidArc)
Image guidance	Daily CBCT

**Table 3:** Organ-at-risk dose constraints for pelvic radiotherapy

Structure	Goal constraint
Rectum	D0.03cc < 64.2 Gy
	V60 < 15%
	V56 < 25%
	V52 < 35%
	V48 < 50%
Bowel space	V45 < 90cc
	D1cc < 55 Gy
	D0.1cc < 60 Gy
Colon	Mean dose
	D2cc
	Maximum dose ≤ 60 Gy
Femoral heads	Mean dose
	D0.03cc
	Maximum dose ≤ 45 Gy

Given the known risk of brain metastases in small-cell bladder carcinoma, PCI was considered but ultimately omitted in favor of MRI surveillance, consistent with institutional practice and available literature. The patient tolerated treatment well with minimal toxicity and continued long-term ADT. Serial imaging and cystoscopic evaluations demonstrated a complete response without evidence of disease recurrence. At the most recent follow-up in 2026, more than three years after completion of therapy, he remains without evidence of disease from either malignancy, with PSA suppressed to 0.13 ng/mL, with a normal PSA range less than or equal to 4 ng/mL, and no intracranial or systemic recurrence.

The patient's clinical course is summarized chronologically as follows: diagnosis of limited-stage SCCB in 2022 after transurethral resection, initiation of neoadjuvant cisplatin/etoposide, development of a necrotizing perineal infection requiring multiple debridements and prolonged rehabilitation, diagnosis of synchronous high-risk prostate adenocarcinoma with initiation of ADT, surgical repair of a large inguinal hernia, completion of definitive SIB-VMAT radiotherapy, and ongoing disease-free follow-up through 2026, as demonstrated in Table 4.

**Table 4:** Chronological timeline of diagnosis, treatment, and follow-up SCCB: small-cell carcinoma of the bladder; PSA: prostate-specific antigen; SIB-VMAT: simultaneous integrated boost-volumetric modulated arc therapy

Timepoint	Clinical event
2022	Presentation with hematuria and urinary retention
2022	Transurethral resection confirming SCCB
2022	Staging investigations demonstrated no evidence of distant metastatic disease
2022	Neoadjuvant cisplatin and etoposide chemotherapy initiated
Following chemotherapy	Necrotizing perineal infection with fistula formation requiring multiple surgical debridements, intravenous antibiotics, and prolonged rehabilitation
During recovery	Markedly elevated PSA (>100 ng/mL) identified
During recovery	Prostate biopsy confirmed synchronous very high-risk prostate adenocarcinoma (Gleason score 4 + 5 = 9)
During recovery	Androgen deprivation therapy initiated
During recovery	Surgical repair of a large inguinal hernia
Approximately 17 months after chemotherapy initiation	Definitive bladder-preserving SIB-VMAT radiotherapy completed
2026	Ongoing complete response without evidence of recurrence; PSA 0.13 ng/mL

## Discussion

Multimodal bladder-preserving therapy

Current National Comprehensive Cancer Network (NCCN) guidelines recommend either concurrent chemoradiotherapy or neoadjuvant systemic therapy followed by definitive local treatment (cystectomy or radiation) for localized SCCB, based primarily on retrospective evidence and extrapolation from SCLC [13]. The median OS is 28.3 months among patients with bladder-confined disease, with comparable outcomes observed between surgery- and radiation-based approaches [1].

Several retrospective series have demonstrated favorable outcomes with combined systemic and local therapy. Multiagent chemotherapy with radiotherapy has achieved five-year OS rates approaching 44%, while neoadjuvant chemotherapy followed by cystectomy has been associated with median OS exceeding 45 months in selected cohorts [21,22]. These findings support the incorporation of platinum-based chemotherapy into the management of localized SCCB.

In the present case, radical cystoprostatectomy was discussed within a multidisciplinary setting but was not favored because of the patient's advanced age, ECOG performance status of 1-2, and substantial comorbidities, including necrotizing perineal infection with fistula formation and a large inguinal hernia requiring repair. The patient therefore proceeded with neoadjuvant cisplatin/etoposide followed by definitive bladder-preserving radiotherapy.

Prior studies have demonstrated substantial treatment responsiveness in SCCB. Neoadjuvant chemotherapy has been associated with high rates of pathologic downstaging and prolonged survival among responders, while bladder-preserving chemoradiation has produced favorable locoregional control with acceptable toxicity profiles [23-25]. These data support bladder preservation as a reasonable option in carefully selected patients who are poor surgical candidates or who desire organ preservation.

Simultaneous treatment of synchronous pelvic malignancies

The concurrent diagnosis of very high-risk prostate adenocarcinoma (Gleason score 4 + 5 = 9, PSA > 100 ng/mL) created a complex therapeutic scenario. Although incidental prostate cancer is commonly identified in cystoprostatectomy specimens, clinically significant synchronous dual pelvic primaries requiring definitive treatment are uncommon [26].

A unified radiation strategy using SIB-VMAT enabled delivery of differential dose levels to the prostate and proximal seminal vesicles, whole bladder, and elective pelvic nodal volumes within a single treatment course. This approach has been successfully applied in other pelvic malignancies and offers favorable target conformity with acceptable toxicity profiles [27].

The bladder dose of 55 Gy in 20 fractions represents a modestly hypofractionated regimen consistent with emerging evidence supporting hypofractionated radiotherapy for bladder cancer. In an individual patient data meta-analysis of the BC2001 and BCON trials, it was demonstrated that hypofractionated schedules achieved locoregional control and toxicity outcomes comparable to conventionally fractionated regimens in locally advanced bladder cancer [28]. Similarly, the prostate dose of 60 Gy in 20 fractions reflects an established moderately hypofractionated regimen for high-risk prostate cancer. The CHHiP trial demonstrated that 60 Gy in 20 fractions achieved disease control and toxicity outcomes comparable to conventionally fractionated 74 Gy in 37 fractions, supporting its adoption as a standard treatment option for localized prostate cancer [29]. Similarly, the PROFIT (Prostate Fractionation Irradiation Trial) prospective, randomized clinical trial found that a moderately hypofractionated regimen of 60 Gy in 20 fractions was well-tolerated and provided similar biochemical control as 78 Gy in 39 fractions for men with intermediate-risk prostate cancer [30]. Furthermore, the PCS5 trial investigated 68 Gy in 25 fractions to the prostate and 45 Gy to the pelvic nodes for high-risk prostate cancer patients and found it to provide equivalent outcomes to 76 Gy in 38 fractions [31].

Accordingly, incorporation of hypofractionated dose schedules within a single integrated treatment plan allowed definitive management of both malignancies while minimizing overall treatment duration and avoiding the need for separate radiation courses. This strategy was particularly advantageous in an elderly patient with substantial comorbidity and prior treatment-related complications. A 24-month ADT was continued for prostate cancer, with sustained PSA suppression observed throughout follow-up.

PCI versus MRI surveillance

The decision to forgo PCI in favor of MRI surveillance in this patient reflects a growing recognition that the natural history of brain metastases in SCCB differs substantially from that of SCLC. In SCLC, PCI is routinely recommended for patients with limited-stage disease who respond to initial therapy, based on evidence from a meta-analysis demonstrating a reduction in brain metastases from 59% to 33% and an associated improvement in OS (HR 0.85) [7].

In contrast, the incidence of brain metastases in SCCB appears to be considerably lower. Bex et al. reported a pooled cumulative incidence of 10.5% (95% CI 7.5%-14.1%) across published SCCB series, markedly below the >50% incidence typically observed in SCLC [18]. Similar findings have been described in extrapulmonary SCC (ESCC), where brain metastases are generally uncommon and routine PCI is not recommended for esophageal, colorectal, small-bowel, appendiceal, or most genitourinary SCCs [15]. Notable exceptions include prostate SCC, in which brain metastases occur in approximately 16%-19% of cases, and head and neck SCC, where rates may approach 41%; in these settings, PCI may be considered selectively [15]. These observations further support the use of MRI surveillance rather than routine PCI in SCCB.

Additional evidence supporting surveillance strategies comes from larger retrospective cohorts. A UK series reported that only six of 409 patients (1.5%) developed brain metastases at any point during follow-up [1]. Likewise, it was also demonstrated that among PCI-eligible patients with M0 ESCC, the three-year cumulative incidence of new brain metastases was only 5.5% [20]. Taken together, these data suggest that the relatively low baseline risk of intracranial relapse in SCCB and EPSCC may not justify the potential neurocognitive toxicity associated with PCI. Consequently, MRI surveillance has emerged as a rational and increasingly favored alternative, allowing for early detection and treatment of intracranial disease while avoiding unnecessary toxicity [17].

In the present case, the patient’s advanced age of 82 years at diagnosis further supported the decision to pursue MRI surveillance rather than PCI. The patient remains disease-free more than three years after treatment, without evidence of intracranial recurrence, which is consistent with the low reported incidence of brain metastases in SCCB and further supports the safety of an MRI surveillance approach in this population.

Treatment delays and intercurrent complications

An unusual aspect of this case was the prolonged interval between initiation of systemic therapy and completion of definitive radiotherapy due to severe necrotizing perineal infection with fistula formation, prolonged rehabilitation, and subsequent surgical repair of a large inguinal hernia. Overall, approximately 17 months elapsed between initiation of chemotherapy and completion of definitive pelvic radiotherapy.

Although prolonged treatment delays are generally associated with inferior outcomes in aggressive malignancies, prior studies suggest that treatment response to neoadjuvant chemotherapy may represent an important prognostic factor in SCCB [24]. Despite the extended delay in definitive local therapy, this patient achieved a complete clinical and radiographic response with durable disease control beyond three years.

Interestingly, the treatment delay also facilitated diagnosis and initiation of therapy for a synchronous very high-risk prostate adenocarcinoma that may otherwise have remained clinically occult at the time of SCCB presentation. Notably, it would have been extremely challenging to subsequently treat his Gleason 4 + 5 = 9 prostate cancer after completion of chemoradiation therapy for his small-cell bladder cancer. While broader conclusions cannot be drawn from a single case, the present report suggests that carefully selected patients with chemosensitive SCCB may still achieve favorable long-term outcomes despite substantial interruptions in definitive local therapy.

A summary of key published studies supporting multimodal therapy, bladder preservation, and central nervous system (CNS) surveillance strategies in SCCB is provided in Table 5.

**Table 5:** A summary of key published studies supporting multimodal therapy, bladder preservation, and CNS surveillance strategies in small-cell carcinoma of the bladder RT: radiotherapy; OS: overall survival; SCCB: small-cell carcinoma of the bladder; TURBT: transurethral resection of bladder tumor; CRT: chemoradiotherapy; DFS: disease-free survival; SCLC: small-cell lung cancer; PCI: prophylactic cranial irradiation; CNS: central nervous system; EPSCC: extrapulmonary small-cell carcinoma; MRI: magnetic resonance imaging

Study	Patients	Treatment approach	Key outcomes	Main conclusion
Cheng et al. (2004) [5]	64	Multimodality (surgery, chemo, RT)	Poor OS; early metastasis common	SCCB is highly aggressive with poor prognosis
Pasquier et al. (2015) [2]	107	Multicenter retrospective	Median OS ~15-29 months overall	Multimodal therapy associated with improved outcomes
Lohrisch et al. (1999) [22]	Single institution	Cisplatin/etoposide + RT	~44% 5-year OS in responders	Long-term survival possible with chemoradiation
Bryant et al. (2016) [25]	18	Chemo + RT after TURBT	High locoregional control (~70%-80%)	Bladder preservation feasible with good control
Chau et al. (2021) [1]	409	UK national cohort	Median OS ~28.3 months (localized disease)	Multimodal therapy improves survival
Bakaloudi et al. (2024) [14]	4,658	Surgery vs. CRT vs. single modality	CRT OS ~29.3 months; surgery + systemic therapy up to ~64 months	Multimodal therapy superior to monotherapy
Teo et al. (2022) [24]	34	Neoadjuvant chemo ± local therapy	Dramatic DFS difference in responders vs. non-responders	Chemotherapy response is prognostic
Bex et al. (2010) [18]	Literature review	SCCB brain metastasis incidence	~10.5% pooled incidence	Brain metastases are uncommon in SCCB
De Caluwé et al. (2017) [20]	51 extrapulmonary SCLC	PCI vs. observation	~5.5% 3-year brain metastasis rate	PCI may not be justified in extrapulmonary disease
Eckert et al. (2012) [32]	Review	CNS outcomes in EPSCC	Low CNS overall failure rates	Supports MRI surveillance strategy
Lopez-Beltran et al. (2017) [26]	Review	Prostate + bladder malignancy coexistence	Incidental prostate cancer common in cystectomy	True synchronous dual primaries are rare

Limitations

This report is limited by its single-patient design, and the favorable outcome may reflect underlying tumor biology and treatment responsiveness rather than generalizable effects of the treatment strategy. The presence of synchronous prostate cancer and concurrent ADT further complicates attribution of outcomes specifically to SCCB-directed therapy. In addition, the absence of prospective data limits definitive conclusions regarding optimal sequencing of therapy, bladder preservation strategies, and the role of PCI omission in SCCB.

## Conclusions

This case illustrates the feasibility of durable long-term disease control using an individualized bladder-preserving multimodal approach in a carefully selected patient with SCCB, substantial comorbidity, prolonged treatment interruption, and synchronous high-grade prostate adenocarcinoma. An SIB radiotherapy strategy incorporating moderately hypofractionated dose schedules permitted definitive treatment of both malignancies within a single pelvic radiation course while maintaining acceptable toxicity.

The absence of intracranial recurrence more than three years after treatment is consistent with emerging evidence suggesting that the risk of brain metastases in SCCB may be lower than that observed in SCLC and that MRI surveillance may represent a reasonable alternative to PCI in selected patients. Although limited by its single-patient nature, this report highlights the importance of individualized multidisciplinary management and demonstrates that favorable long-term outcomes may be achievable in clinically complex presentations of SCCB. Further study is needed to better define optimal treatment strategies, patient selection, and CNS surveillance approaches in this rare disease.
